# Emergency Department Placed Central Lines for Trauma Patients: A Retrospective Case-Control Study on Central Line–Associated Blood Stream Infection Risk From Central Lines Placed Emergently in the Emergency Department

**DOI:** 10.1016/j.acepjo.2025.100047

**Published:** 2025-02-13

**Authors:** Larissa Epstein, Jeffry Nahmias, Sebastian Schubl, Kenji Inaba, Kazuhide Matsushima, Michael Lekawa, Matthew Dolich, Areg Grigorian

**Affiliations:** 1 Department of Surgery, University of Southern California, Los Angeles, California, USA; 2 Division of Trauma, Burns and Surgical Critical Care, Department of Surgery, University of California, Irvine Medical Center, Orange, California, USA

**Keywords:** CLABSI, emergency department, central line, central venous catheter

## Abstract

**Objectives:**

Emergent central line (CL) insertion may be associated with a higher risk of central line–associated blood stream infection (CLABSI). We hypothesized that CLs placed emergently within 2 hours of arrival to the emergency department (ED) for critical trauma patients are associated with a higher risk of CLABSI compared with CLs placed outside the ED. We additionally hypothesized that femoral ED-CLs are associated with a higher risk of CLABSI compared with internal jugular (IJ) vein ED-CLs.

**Methods:**

The 2017-2019 Trauma Quality Improvement Program database was queried for critical trauma patients admitted to the intensive care unit or operating suite from the ED who underwent CL insertion. Patients who were transferred, died < 72 hours, or hospitalized <2 days were excluded. A total of 27,981 patients met inclusion criteria and 169 of these patients met criteria for a CLABSI. Patients receiving an ED-CL within 2 hours of arrival were compared with patients receiving a CL outside of the ED (non-ED-CL). We performed a subanalysis of only ED-CL patients for risk of CLABSI dependent on insertion site. A multivariable logistic regression analysis was performed.

**Results:**

Of 27,981 patients, 7908 (28.3%) received an ED-CL mostly in the subclavian vein (51.5%). After adjusting for risk factors, ED-CL patients had a similar risk of CLABSI (odds ratio [OR], 0.75; CI, 0.51-1.11; *P* = .15), compared with non-ED-CL patients. Among ED-CL patients, insertion of a subclavian CL (OR, 0.40; CI, 0.18-0.87; *P* = .02) was associated with a lower risk of CLABSI compared with an IJ CL, whereas femoral and IJ CLs had a statistically nonsignificant difference in risk of CLABSI (OR, 0.46; CI, 0.20-1.05; *P* = .06).

**Conclusion:**

Insertion of ED-CLs within 2 hours of arrival is not associated with a higher risk of CLABSI compared with insertion of a non-ED-CLs. The subclavian vein is the most common site for emergent CL insertion in the ED. For ED-CLs, the subclavian line is associated with the lowest risk of CLABSI and should be considered the optimal site for insertion in critically ill trauma patients with no known history of chronic kidney disease.

**The Bottom Line**

The belief among intuitions is that emergently placed central lines are associated with higher rates of central line blood infections due to compromise in sterile technique; however, this dogma has never been studied. Commonly, emergently placed central lines will be replaced in controlled settings once patients reach their final destination in the hospital, but replacing central lines is not without the risks of pneumothoraces, hemothoraces, etc. This retrospective study suggests that critical trauma patients who receive an emergent central line are not at increased risk for bloodstream infections which challenges the belief that central lines must be exchanged if placed emergently.

## 1 Introduction

### 1.1 Background

A central line (CL) is often required for critically ill patients to provide venous access when peripheral access cannot be obtained or allows for infusion of potent medications, rapid transfusion of blood products, and monitoring of central pressures.^1^^,^^2^ The subclavian, femoral, and internal jugular (IJ) veins are all locations for central catheterization with the IJ and subclavian being the most common sites; however, this is largely institution dependent.^3^ CL insertion can lead to complications such as pneumothorax, hemothorax, vascular injuries, central line–associated blood stream infection (CLABSI), deep vein thrombosis (DVT), air embolus, and pulmonary embolus (PE).^4^

### 1.2 Importance

The Centers for Disease Control and Prevention (CDC) defines CLABSI as bacteremia occurring in a patient with a CL or a patient with a CL removed in the previous 48 hours and without any other source of infection identified.^5^ Patients with CLABSI have a 25% increased risk of mortality and over a 35% increased risk of readmission.^6^, ^7^, ^8^ CLABSI also leads to increased hospital length of stay (LOS) and resource utilization, costing nearly $2 billion in medical costs and lost revenue annually.^9^

Current guidelines including the American Society of Anesthesiologists Task Force and the CDC recommend the insertion of upper extremity CLs as the preferred site due to a presumed lower risk of complications including CLABSI.^10^^,^^11^ Historically, the femoral vein has been considered to have the higher risk of infections due to the proximity to the groin.^10^ With antiseptic techniques and maintenance of CLs after insertion, this view has been brought into question.^12^, ^13^, ^14^ A prospective observational study in critically ill patients by Deshpande et al^12^ demonstrated no difference in CLABSI risk between any of the main sites of CL insertion. However, this study and the existing literature focus mainly on medical patients.^12^ Trauma patients are unique as they may present in extremis requiring emergent CL insertion performed in the emergency department (ED). There exists institutional bias among CLs placed in the ED with trauma centers exchanging all CLs placed in the ED within 48 hours due to the perception that sterile technique is compromised during emergent CL insertion and the fear of potential infectious complications.^15^ This is based on category IB evidence.^11^ This practice has never been demonstrated to improve outcomes and CL exchange is not without complications.^4^^,^^14^ Outcomes for CLs placed in the ED for critically ill trauma patients have not been previously studied using a large national sample. In fact, the National Healthcare Safety Network, the CDC’s National Healthcare Infection Tracking System, does not even track CLABSI in the ED because the ED is not considered an inpatient unit.^15^ There have been single institutional studies comparing ED-placed CLs with CLs placed in the intensive care unit (ICU) CLABSI rates. Both Theodoro et al^9^ and Inhofer et al^16^ showed the CLABSI rates of those CLs placed in the ED were within the range of those placed in the ICU. Although these studies were not specific toward trauma patients, they call into question this archaic policy of line exchanges in ED-placed CLs.

### 1.3 Goals of This Investigation

This study aimed to compare CL insertion in the ED with non-ED locations and evaluate the insertion site as a risk factor for CLABSI in ED-placed CLs. We hypothesized that CLs placed emergently in the ED within 2 hours of arrival for critically ill trauma patients are associated with a higher risk of CLABSI compared with CLs placed outside of the ED. We additionally hypothesized that ED-placed femoral CLs are associated with a higher risk of CLABSI compared with ED-placed IJ vein CLs.

## 2 Methods

### 2.1 Design and Setting

This is an observational retrospective case-control study. The Strengthening the Reporting of Observational Studies in Epidemiology (STROBE) guidelines were followed.^17^ The 2017-2019 Trauma Quality Improvement Program (TQIP) database was queried for critically ill trauma patients admitted to either the ICU or operating suite (OS) from the ED, who underwent CL insertion. TQIP began in 2008 by the American College of Surgeons Committee on Trauma as a counterpart to the American College of Surgeons National Surgical Quality Improvement Program.^18^ There are criteria for eligibility among the various levels in TQIP but for our paper’s purposes, the data obtained come from verified level 1 and level 2 trauma centers, those provisionally designated as a level 1 or 2 trauma center, and those that have applied as a level 1 or 2 trauma center and are currently undesignated. The database is maintained by the American College of Surgeons with more than 900 trauma centers across the United States participating. A fee is required to view the database online; however, institutions participating in TQIP are exempt from this fee. TQIP provides risk adjusted data to reduce variability in adult trauma outcomes for research purposes as well as provide best practice guidelines to improve trauma care and outcomes.^18^ A multicenter study in the *Journal of the American Medical Association* (*JAMA*) has demonstrated that hospitals that participate in regional collaborative quality improvement programs are associated with improved patient outcomes.^18^ TQIP codes CLs on anatomic location of the lines, the physical location of the patient when the lines are placed (ie, ED, OS, and surgical ICU), and how soon after admission the CLs are placed, among other characteristics.

### 2.2 Selection of Participants

The institutional review board deemed this study exempt and granted a consent waiver because the data used are from a national deidentified database. Exclusions included patients who were transferred from outside hospitals, died within 72 hours, or with an LOS <48 hours. Patients who received a central line in the ED (ED-CL) within 2 hours of arrival were then compared with patients who received a CL in any location other than the ED and not within a 2-hour time limit (non-ED-CL). The 2-hour cutoff in the ED was guided by TQIP, which records all CL placements on an hourly basis. Patients receiving CLs within the first hour of arrival are typically among the most critically ill and necessitate immediate intervention. However, there is a subset of patients who, despite presenting in stable condition, experience deterioration after the first hour due to the severity of their injuries. This often mandates ED-CL placement as they await transfer to another unit or their final hospital destination. By limiting the study to only those receiving a CL within the first hour of ED arrival, there would be an excluded cohort who, while not requiring immediate intervention, still represented cases of emergent CL placement. To ensure the study captures this crucial patient demographic, we opted for the 2-hour window. We performed a power calculation and to observe a 2% difference in the rate of CLABSI between the groups we would need at least 768 patients in each group for a beta value of 0.8.

### 2.3 Outcomes

The primary outcome was CLABSI, defined in Supplementary Appendix 1. A CLABSI is defined as a laboratory-confirmed bloodstream infection with a catheter in place for at least 2 days. The CL must have been in place during the inciting event or removed no more than 1 day prior, and the pathogens may not be related to an infection elsewhere in the body. We additionally performed a subset analysis comparing risk of CLABSI for different anatomic sites in ED-CL patients. The control group for this subanalysis was the IJ location.

### 2.4 Measurements

Demographic variables including age, sex, and comorbidities were collected. Comorbidities included congestive heart failure, cirrhosis, chronic obstructive pulmonary disease, diabetes, hypertension, smoking, steroid use, and myocardial infarction (MI). The injury profile included trauma mechanism, Injury Severity Score (ISS), vitals on admission including hypotension (systolic blood pressure [SBP] <90 mm Hg), tachycardia (>120 beats/min), tachypnea (respiratory rate >22 breaths/min), as well as specific injuries anatomic sites as seen in Table 1. We collected the rates of additional in-hospital complications other than CLABSI including cardiac arrest, catheter-associated urinary tract infections, deep surgical site infection (SSI), DVT, PE, unplanned intubation, acute kidney injury, MI, organ space SSI, osteomyelitis, pressure ulcer, acute respiratory distress syndrome, unplanned return to the OS, sepsis, stroke, superficial incisional SSI, unplanned admission to the ICU, ventilator-associated pneumonia, and death.

**Table 1:** Demographics and injury profile of patients with central lines placed in ED vs non-ED.

Characteristic	ED-CL	Non-ED-CL	% Difference	*P* value
	(n = 7908)	(n = 20,073)		
Age (y), median (IQR)	40 (26, 55)	50 (34, 67)	10	<.001
Male, n (%)	5886 (74.4%)	14,525 (72.4%)	2.0%	<.001
Mechanism, n (%)				
Blunt	5558 (70.3%)	16,804 (83.7%)	–13.4%	<.001
Penetrating	2238 (28.3%)	2599 (12.9%)	15.4%	<.001
Comorbidities, n (%)				
Congestive heart failure	141 (1.8%)	908 (4.6%)	–2.8%	<.001
Cirrhosis	102 (1.3%)	457 (2.3%)	–1.0%	<.001
COPD	264 (3.4%)	1336 (6.7%)	–3.3%	<.001
Diabetes	610 (7.9%)	2836 (14.3%)	–6.4%	<.001
Hypertension	1390 (17.9%)	6089 (30.7%)	–12.8%	<.001
Smoker	1619 (20.9%)	4162 (21.0%)	–0.1%	.809
Steroid use	153 (0.8%)	37 (0.5%)	0.3%	.008
Myocardial infarction	36 (0.5%)	191 (1.0%)	–0.5%	<.001
Vitals, n (%)				
Hypotensive (SBP < 90 mm Hg)	2105 (27.3%)	3241 (16.4%)	10.9%	<.001
Tachycardia > 120 (beats/min)	2521 (32.2%)	4277 (21.6%)	10.6%	<.001
Respiratory rate > 22 (breaths/min)	2856 (37.8%)	6526 (34.0%)	3.8%	<.001
ISS > 25, n (%)	4146 (52.5%)	10,836 (46.0%)	6.5%	<.001
Injury, n (%)				
Liver	1357 (17.2%)	2263 (11.3%)	5.9%	<.001
Kidney	636 (8.0%)	1263 (6.3%)	1.7%	<.001
Spleen	1188 (15.0%)	2251 (11.2%)	3.8%	<.001
Stomach	224 (2.8%)	321 (1.6%)	1.2%	<.001
Pancreas	211 (2.7%)	396 (2.0%)	0.7%	<.001
Small intestine	723 (9.1%)	1132 (5.6%)	3.5%	<.001
Colon	660 (8.3%)	1099 (5.5%)	2.8%	<.001
Rectum	64 (0.8%)	130 (0.6%)	0.2%	.142
Hemothorax	595 (7.5%)	1232 (6.1%)	1.4%	<.001
Pneumothorax	1927 (24.4%)	4328 (21.6%)	2.8%	<.001
Lung	2422 (30.6%)	5183 (25.8%)	4.8%	<.001
Hemopneumothorax	1167 (14.8%)	2008 (10.0%)	4.8%	<.001
Rib	3263 (41.3%)	8194 (40.8%)	0.5%	.499
Heart	291 (3.7%)	488 (2.4%)	1.3%	<.001
Thoracic vessels	339 (4.3%)	632 (3.1%)	1.2%	<.001
Femur	1097 (13.9%)	2799 (13.9%)	0.0%	.875
Humerus	727 (9.2%)	1632 (8.1%)	1.1%	.004
Fibula	851 (10.8%)	1977 (9.8%)	1.0%	.023
Tibia	1035 (13.1%)	2399 (12.0%)	1.1%	.009
Lower extremity nerve	30 (0.4%)	73 (0.4%)	0.0%	.845
Upper extremity nerve	156 (2.0%)	225 (1.1%)	1.1%	<.001

COPD, chronic obstructive pulmonary disease; ED-CL, central line placed in the emergency department within 2 hours; ISS, Injury Severity Score; non-ED-CL, central line placed outside of the emergency department; SBP, systolic blood pressure.

### 2.5 Analysis

A Mann-Whitney U test was used to compare continuous variables, whereas a chi-squared test was performed to compare categoric data. Continuous data were reported as median values with an IQR, and categoric data were reported as percentages.

The risk of CLABSI was measured using a multivariable logistic regression analysis. The association between predictor variables and the incidence of CLABSI was measured using a univariable logistic regression model. The variables, which were coded by TQIP, were chosen, a priori, after a discussion among coauthors and review of the literature to identify risk factors for CLABSI that are available in TQIP.^19^^,^^20^ Covariates (hypotension, tachycardia, tachypnea, packed red blood cell transfusion, surgical intervention, ISS, diabetes, smoking, and steroid use) were then entered into a multivariable logistic regression model, and the adjusted risk for CLABSI was reported with an odds ratio (OR) and 95% CI. All *P* values had a statistical significance level of <.05. All statistical analyses were performed using IBM SPSS Statistics for Windows (Version 28, IBM Corp).

## 3 Results

### 3.1 Characteristics of Study Subjects

Patient demographics are shown in Table 1. The median age, percentage female, percentage penetrating trauma, percentage with hypotension, and percentage with an ISS >25 for the ED-CL and non-ED-CL cohort, respectively, are as follows: 40 and 50, 25.6% and 27.6%, 28.3% and 25.9%, 27.3% and 16.4%, 52.5% and 46.0%. All demographics between the 2 groups other than smoking were statistically significant (*P* < .001). In addition, more patients with ED-CLs had unstable vital signs within the 2 hours of being in the ED as well as a higher percentage of ISS >25.

Of 27,981 patients, 7908 (28.3%) received an ED-CL within 2 hours of arrival (Fig). The remaining 20,073 (71.7%) patients received a non-ED-CL. Compared with the non-ED-CL group, ED-CL patients were younger (median age in years, 40 vs 50, *P* < .001) and had a higher rate of penetrating trauma (28.3% vs 12.9%, *P* < .001), and hypotension on arrival (27.3% vs 16.4%, *P* < .001) (Table 2).

**Figure:**
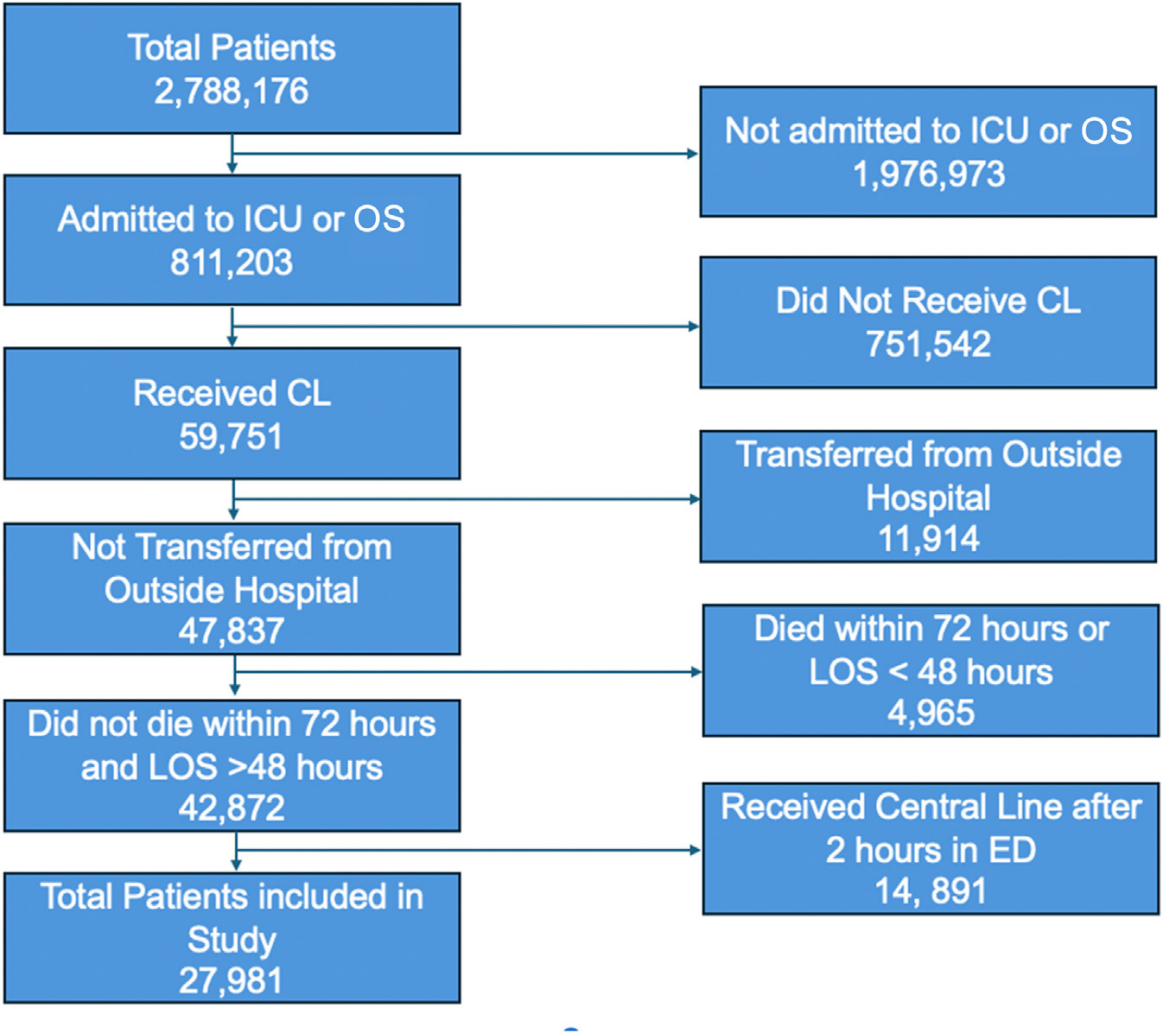
Flow diagram for inclusion criteria of study. CL, central line; ED, emergency department; ICU, intensive care unit; LOS, length of stay; OS, operating suite.

**Table 2:** Complications obtained by patients with central lines placed in ED vs non-ED.

Complication, n (%)	ED-CL	Non-ED-CL	% Difference	*P* value
	(n = 7908)	(n = 20,073)		
Cardiac arrest	503 (6.4%)	1352 (6.8%)	–0.4%	.267
CAUTI	110 (1.4%)	406 (2.0%)	–0.6%	<.001
CLABSI	43 (0.5%)	126 (0.6%)	–0.1%	.418
Deep SSI	77 (1.0%)	183 (0.9%)	0.1%	.622
Deep vein thrombosis	483 (6.1%)	1246 (6.2%)	–0.1%	.768
Pulmonary embolism	159 (2.0%)	445 (2.2%)	–0.2%	.290
Unplanned intubation	323 (4.1%)	2182 (10.9%)	–6.8%	<.001
Acute kidney injury	410 (5.2%)	1505 (7.5%)	–2.3%	<.001
Myocardial infarction	45 (0.6%)	233 (1.2%)	–0.6%	<.001
Organ space SSI	87 (1.1%)	161 (0.8%)	0.3%	.016
Osteomyelitis	13 (0.2%)	39 (0.2%)	0.0%	.604
Pressure ulcer	278 (3.5%)	986 (4.9%)	–1.4%	<.001
ARDS	249 (3.2%)	935 (4.7%)	–1.5%	<.001
Unplanned return to OS	405 (5.4%)	908 (4.8%)	0.6%	.042
Sepsis	171 (2.2%)	835 (4.2%)	–2.0%	<.001
Stroke	138 (1.8%)	469 (2.3%)	–0.5%	.002
Superficial incisional SSI	73 (0.9%)	169 (0.8%)	0.1%	.504
Unplanned admission to ICU	309 (3.9%)	1462 (7.3%)	–3.4%	<.001
VAP	397 (5.0%)	1498 (7.5%)	–2.5%	<.001
Death	1681 (21.3%)	4338 (21.6%)	–0.3%	.516

ARDS, acute respiratory distress syndrome; CAUTI, catheter-associated urinary tract infection; CLABSI, central line–associated blood stream infection; ICU, intensive care unit; SSI, surgical site infection; OS, operating suite; VAP, ventilatory-associated pneumonia.

### 3.2 Main Results

The overall rate of CLABSI in the study population was 0.6% (n = 169). There was no significant difference in CLABSI rate between both groups (ED-CL 0.5%, non-ED-CL 0.6%, *P* = .418) (Table 3).

**Table 3:** Univariate logistic regression analysis for risk of CLABSI for central lines.

Characteristic	OR	CI	*P* value
Hypotension (SBP < 90 mm Hg)	1.53	1.10-2.13	.013
Tachycardia > 120 (beats/min)	1.92	1.44-2.56	<.001
Respiratory rate > 22 (breaths/min)	1.11	0.82-1.51	.481
PRBC transfusion	1.81	1.38-2.38	<.001
Surgical intervention	6.52	3.85-11.02	<.001
ISS categories (reference < 9)			<.001
9-15	1 1.22	0.60-2.47	.582
16-25	2.63	1.51-4.61	<.001
>25	3.96	2.35.-6.66	<.001
Diabetes	0.92	0.60-1.41	.702
Smoking	0.76	0.53-1.08	.127
Steroid use	0.00	0.00	.995

CLABSI, central line–associated blood steam infection; ED-CL, central line placed in the emergency department within 2 hours; ISS, Injury Severity Score; non-ED-CL, central line placed outside of the emergency department; OR, odds ratio; PRBC, packed red blood cell; SBP, systolic blood pressure.

After adjustment, there remained no difference in the associated risk of CLABSI between ED-CL and non-ED-CL patients (OR, 0.75; CI, 0.51-1.1; *P* = .151) (Table 4).

**Table 4:** Multivariable logistic regression analysis for risk of CLABSI for central line placed in ED vs non-ED.

Characteristic	OR	CI	*P* value
ED-CL vs non-ED-CL	0.75	0.51-1.11	.151
Hypotension (SBP < 90 mm Hg)	1.05	0.69-1.59	.834
Tachycardia > 120 (beats/min)	1.60	1.12-2.30	.010
Respiratory rate > 22 (breaths/min)	0.82	0.58-1.17	.274
PRBC transfusion	1.02	0.71-1.47	.931
Surgical intervention	5.93	2.89-12.15	<.001
ISS categories (reference < 9)			.100
9-15	1.48	0.58-3.78	.410
16-25	2.13	0.95-4.77	.068
>25	2.36	1.08-5.17	.031
Diabetes	0.94	0.55-1.62	.831
Smoking	0.71	0.46-1.11	.136
Steroid use	0.00	0.00	.996

CLABSI, central line–associated blood steam infection; ED-CL, central line placed in the emergency department within 2 hours; ISS, Injury Severity Score; non-ED-CL, central line placed outside of the emergency department; OR, odds ratio; PRBC, packed red blood cell; SBP, systolic blood pressure.

Among the 7908 ED-CL patients, 4075 (51.5%) had a subclavian CL insertion. Among ED-CL patients, insertion of a subclavian CL was associated with a lower rate of CLABSI (0.4% vs 1.0%, *P* = .015) when compared with IJ CL (OR, 0.40; CI, 0.18-0.87; *P* = .021) (Table 5). Femoral ED-CLs had a lower rate of CLABSI (0.4% vs 1.0%, *P* = .019) compared with IJ CLs but after adjustment, the risk of CLABSI was similar (OR, 0.46; CI, 0.20-1.04; *P* = .063) (Table 6).

**Table 5:** Multivariable logistic regression analysis for risk of CLABSI for subclavian lines vs IJ lines.

Characteristic	OR	CI	*P* value
Subclavian vs IJ lines	0.40	0.18-0.87	.021
Subclavian lines (n): 4075			
IJ lines (n): 1358			
Hypotension (SBP < 90 mm Hg)	1.41	0.60-3.30	.429
Tachycardia > 120 (beats/min)	2.04	0.90-4.63	.087
Respiratory rate > 22 (breaths/min)	0.70	0.30-1.62	.405
PRBC transfusion	0.91	0.36-2.29	.846
Surgical intervention	2.89	0.66-12.55	.157
ISS categories (reference < 9)			.799
9-15	0.00	0.00	.993
16-25	0.84	0.16-4.46	.836
>25	1.37	0.30-6.20	.681
Diabetes	0.59	0.08-4.44	.611
Smoking	0.93	0.35-2.58	.879
Steroid use	0.00	0.00	.999

CLABSI, central line–associated blood steam infection; ED-CL, central line placed in the emergency department within 2 hours; IJ, internal jugular; ISS, Injury Severity Score; non-ED-CL, central line placed outside of the emergency department; OR, odds ratio; PRBC, packed red blood cell; SBP, systolic blood pressure.

**Table 6:** Multivariable logistic regression analysis for risk of CLABSI for femoral lines vs IJ lines.

Characteristic	OR	CI	*P* value
Femoral vs IJ lines	0.46	0.20-1.04	.063
Femoral lines (n): 3248			
IJ lines (n): 1358			
Hypotension (SBP < 90 mm Hg)	0.84	0.32-2.21	.722
Tachycardia > 120 (beats/min)	1.83	0.77-4.32	.170
Respiratory rate > 22 (breaths/min)	1.38	0.59-3.21	.453
PRBC transfused	0.81	0.31-2.11	.662
Surgical intervention	0.00	0.00	.990
ISS categories (reference < 9)			.451
9-15	0.43	0.04-4.77	.488
16-25	0.68	0.12-3.79	.655
>25	1.38	0.31-6.26	.674
Diabetes	0.62	0.08-4.70	.646
Smoking	1.69	0.69-4.15	.250
Steroid use	0.00	0.00	.998

CLABSI, central line–associated blood steam infection; ED-CL, central line placed in the emergency department within 2 hours; IJ, internal jugular; ISS, Injury Severity Score; non-ED-CL, central line placed outside of the emergency department; OR, odds ratio; PRBC, packed red blood cell; SBP, systolic blood pressure.

## 4 Limitations

This study has several limitations, including its retrospective observational design, which precludes us from making any conclusions about causation. Data were collected from a large national database, which is prone to coding errors and reporting bias. The database lacks granular data related to the actual CL placement, such as the use of sterile technique, ultrasound guidance, number of attempts, adherence to a CLABSI prevention bundle, and the provider performing the procedure’s training level.

We are also unable to randomize the placement of the ED-CLs which leads to a form of confounding by indication. Additionally, our study did not capture data related to patient-specific factors that may have affected the risk of infection, such as immunocompromised state, poor nutritional status, and duration of antibiotics.^21^^,^^22^ Moreover, our study did not account for CL care after insertion, which may have impacted the risk of CLABSI. Also, our findings may be subject to selection bias, as certain institutions may have specific protocols or preferences for CL insertion sites, nor did we include patients with CLs placed in the ED and then admitted to the regular floor because our emphasis was on critically ill trauma patients. Finally, the data were not analyzed based on catheter days, which has a strong association with risk of infection.^23^^,^^24^

In addition, 27,891 patients were included in our study. Of this large sample size, only 169 patients were confirmed CLABSI cases, or 0.6% of our sample size. This small sample size may limit our ability to detect smaller differences between groups.

These limitations should be considered when interpreting the results of this study and highlight the need for further prospective research on CLABSI in trauma patients.

## 5 Discussion

This study challenges the dogma that ED-CLs are prone to infection due to being placed emergently in unstable patients, which leads providers to perhaps question the need to urgently replace these CLs in a more controlled and sterile setting.

Our study focuses specifically on trauma patients, whereas much of the existing literature has reported on medical patients. This analysis demonstrates that the CLABSI rate has decreased in the modern era, with <1% of critically ill patients developing this complication. Interestingly, our data suggest a CLABSI rate of 0.6%, whereas nationally the CLABSI rate is slightly higher at 0.9% in 2021.^25^ Although we cannot comment on the reasoning behind this, we postulate that this may be due to the younger and healthier population who are often victims of trauma. Also, the rate of any CL insertion (at any point in time) for trauma patients with ISS >25 was 15.5% (n = 28,072). Our findings suggest that critically ill trauma patients who receive an ED-CL are not at higher risk of CLABSI compared with those who receive a non-ED-CL. This is reassuring, given the perceived potential risk for compromised sterile technique in the emergent ED setting. Among patients with ED-CLs, the subclavian vein appears to be the most commonly chosen insertion site and has the lowest risk of CLABSI. These results can inform the choice of insertion site and management of CLs in trauma patients, with the ultimate goal of improving patient safety and reducing nosocomial infections.

The rate of CLABSI has decreased over the past several decades. The CDC reported that the rate of CLABSI has decreased by more than half from 2008 to 2016, and by 2020, it was reduced by an additional 50%.^26^^,^^27^ The use of CL bundles, which includes hand hygiene, emphasis on antiseptic techniques, maximal sterile barrier precautions, chlorhexidine skin preparation, and site selection with a multidisciplinary approach, is likely a significant contributor to this.^28^, ^29^, ^30^, ^31^, ^32^ These measures have significant implications, given that up to 40% of critically ill patients in the hospital have a CL.^33^ Our study found a low CLABSI rate (<1%) even among critically ill trauma patients with ED-CLs, suggesting that factors related to initial insertion (such as patient condition, emergent nature, or provider experience) may be mitigated with attention to proper technique, routine CL care, and timely removal of CLs.^9^

The physical location of CL insertion has long been postulated to be associated with the risk of CLABSI, with surgical dogma suggesting that CLs placed in the ED have a higher CLABSI risk compared with CLs placed elsewhere in the hospital.^34^ However, this belief has not been supported, and a previous single-center retrospective analysis found no difference in the CLABSI rate between ED-placed CLs and those placed in the ICU, as long as sterile technique was used.^16^ Our study builds on this research by including a national sample of patients undergoing emergent CL insertion in the ED. Although we were unable to determine the use of sterile technique for these patients, our findings refute the notion that ED-placed CLs must be exchanged within 24 hours and suggest that the routine practice of exchanging ED-placed CLs may not be necessary and only associated with increased cost and resource use.

The anatomic location of CL insertion may also be related to the risk of CLABSI. The subclavian vein has been shown to be associated with the lowest CLABSI risk and is considered the preferred initial location site by various societal guidelines.^11^^,^^35^^,^^36^ This may be due to decreased colonization rates for subclavian vein sites when compared with IJ and femoral vein sites.^37^ Our study corroborates prior reports that the subclavian vein is associated with a lower CLABSI risk compared with femoral and IJ locations, even among emergently placed CLs in the ED.^38^ Although we found no statistically significant difference in the risk of CLABSI between femoral and IJ placed CLs in the ED, there was a trend toward a lower risk of CLABSI with femoral vein placement. This finding is supported by several meta-analyses and multicenter prospective studies.^13^^,^^39^^,^^40^ A study by Gowardman et al^41^ illustrated that although the femoral site has an increased rate of colonization, the infection rate between all 3 sites remains similar. Nonetheless, we recommend the subclavian vein as the preferred site for CL insertion in the ED, unless there is a contraindication, such as chronic kidney disease or concern for central vascular injury near this region. This may help further reduce the risk of CLABSI and improve patient outcomes. It is important to note that site selection should be individualized based on patient-specific factors and clinical judgment.

This national analysis spanning 3 years of data found that the subclavian vein is the most commonly used site for emergent ED-placed CLs and is associated with a lower CLABSI risk compared with femoral CL insertions. Our data also show that subclavian ED-CLs have a statistically significant lower CLABSI rate when compared with IJ ED-CLs. In addition, femoral and IJ ED-CLs did not show a difference in CLABSI rate. Based on these findings in conjunction with our results, we continue to recommend the subclavian vein as the optimal site for CL insertion in most critically ill trauma patients even with more recent meta-analyses bringing this teaching into question. It is important to acknowledge that subclavian CLs have risks with placement. In addition, insertion of any CL in the ED within 2 hours of arrival is not associated with a higher CLABSI risk compared with insertion of a CL outside of the ED. This challenges existing surgical dogma that all ED-placed CLs should be routinely exchanged.

## Author Contributions

All authors contributed to data retrieval and editing of the manuscript. Data were analyzed by Dr Grigorian. The manuscript was primary written by Dr Epstein. Dr Grigorian serves as the principle investigator.

## Funding and Support

By *JACEP Open* policy, all authors are required to disclose any and all commercial, financial, and other relationships in any way related to the subject of this article as per ICMJE conflict of interest guidelines (see www.icmje.org). The authors have stated that no such relationships exist.

## Conflict of Interest

All authors have affirmed they have no conflicts of interest to declare.
