# Supplementary material for: Emergency Department Placed Central Lines for Trauma Patients: A Retrospective Case-Control Study on Central Line–Associated Blood Stream Infection Risk From Central Lines Placed Emergently in the Emergency Department
Source: J Am Coll Emerg Physicians Open. 2025 Feb 13;6(2):100047. doi: 10.1016/j.acepjo.2025.100047 (PMC11874560; doi:10.1016/j.acepjo.2025.100047)
Supplement: Supplementary Appendix 1 [file mmc1.docx]

**APPENDIX A:**

| **CLABSI was defined as meeting all the following criteria from the National Trauma Data Standard Data Dictionary:** | | |
| --- | --- | --- |
| Laboratory confirmed blood stream infection in one or more blood specimens |  |  |
| CL in place for >2 days (day of placement is day 1) |  |  |
| CL in place during inciting event | OR | CL removed no more than one day prior |
| Pathogens in blood are not related to an infection at another site |  |  |
| AND/OR at least one of the following signs: hypotension, fever, chills |  |  |
